# Modulating the Actin Cytoskeleton Affects Mechanically Induced Signal Transduction and Differentiation in Mesenchymal Stem Cells

**DOI:** 10.1371/journal.pone.0071283

**Published:** 2013-07-29

**Authors:** Petra Müller, Anne Langenbach, Alexander Kaminski, Joachim Rychly

**Affiliations:** 1 Laboratory of Cell Biology, Rostock University Medical Center, Rostock, Germany; 2 Department of Heart Surgery, Rostock University Medical Center, Rostock, Germany; Dalhousie University, Canada

## Abstract

Mechanical interactions of mesenchymal stem cells (MSC) with the environment play a significant role in controlling the diverse biological functions of these cells. Mechanical forces are transduced by integrins to the actin cytoskeleton that functions as a scaffold to switch mechanical signals into biochemical pathways. To explore the significance of cytoskeletal mechanisms in human MSC we modulated the actin cytoskeleton using the depolymerising drugs cytochalasin D (CytD) and latrunculin A (LatA), as well as the stabilizing drug jasplakinolide (Jasp) and examined the activation of the signalling molecules ERK and AKT during mechanical loading. All three drugs provoked significant changes in cell morphology and organisation of the cytoskeleton. Application of mechanical forces to β1-integrin receptors using magnetic beads without deformation of the cell shape induced a phosphorylation of ERK and AKT. Of the two drugs that inhibited the cytoskeletal polymerization, LatA completely blocked the activation of ERK and AKT due to mechanical forces, whereas CytD inhibited the activation of AKT but not of ERK. Activation of both signalling molecules by integrin loading was not affected due to cell treatment with the cytoskeleton stabilizing drug Jasp. To correlate the effects of the drugs on mechanically induced activation of AKT and ERK with parameters of MSC differentiation, we studied ALP activity as a marker for osteogenic differentiation and examined the uptake of fat droplets as marker for adipogenic differentiation in the presence of the drugs. All three drugs inhibited ALP activity of MSC in osteogenic differentiation medium. Adipogenic differentiation was enhanced by CytD and Jasp, but not by LatA. The results indicate that modulation of the cytoskeleton using perturbing drugs can differentially modify both mechanically induced signal transduction and MSC differentiation. In addition to activation of the signalling molecules ERK and AKT, other cytoskeletal mechanisms are involved in MSC differentiation.

## Introduction

Mechanical forces in the microenvironment of adult stem cells play a decisive role in controlling the fate of these cells [1]–[4]. Within the tissues stem cells are constantly subjected to external forces and are able to adjust to their changes. The forces that are required to regulate the differentiation of mesenchymal stem cells (MSC) to multiple lineages correlate with the mechanical properties of the specific tissue [5]. Both 2D *in vitro* systems as well as 3D experiments demonstrated that soft matrix promoted fat cell differentiation whereas a rigid substrate facilitates osteogenic differentiation [5], [6]. Similarly, to maintain stem cells in the state of pluripotency and self-renewal a defined mechanical environment is required [7]. The main cellular components that mediate mechanical forces from the extracellular matrix outside the cells into the cell interior are integrin receptors that bind to proteins of the extracellular matrix and are able to transmit forces by physical interacting with the actin cytoskeleton [8]–[10]. The backbone of the cytoskeleton is F-actin, which clusters to form filaments. The filaments can be bundled and cross-linked by actin-binding proteins to form a network [11]. This actin filamentous network is highly dynamic. Cells are able to sense the mechanical properties of the adhesive substrate through a balance between the cytoskeletal contractibility facilitated by actomyosin and the resistant forces of the extracellular matrix [12], [13]. The dynamic behaviour of the actin cytoskeleton forms the basis for a number of cellular functions including migration or division [14]. With the progress in stem cell research it became obvious that the actin cytoskeleton is a central modulator that controls function and modulates differentiation [15]. The structural organization of the cytoskeletal network determines the cell shape which was found to regulate the fate of stem cells. Evidence exists that differentiation to chondrocytes requires a more rounded phenotype which can be facilitated by a pellet culture or encapsulation of the cells [16], [17]. When used the technique of micropatterning, round MSC differentiated to adipocytes, whereas spread cells developed to osteoblasts [18]. In addition to sensing mechanical forces, the cytoskeleton forms a structure to transform mechanical forces into biochemical signals. Due to the contractibility of the actin filaments, proteins associated with the cytoskeleton may be stretched which results in an unfolding and presenting of new binding sites [19]. Such mechanisms can lead to an activation of signalling proteins by phosphorylation. In addition, forces can be transduced from the cell surface to the nucleus via the actin cytoskeleton by a direct mechanocoupling [20]. This process propagates the mechanical signal much faster through the cytoplasm and induces biochemical events in the nucleus. Despite the central role of the actin cytoskeleton in mechanically induced signalling and biological responses in mesenchymal stem cells, little is known about the effects of modulation of the actin cytoskeleton in these cells by known drugs that impair or stabilize actin polymerization. We demonstrate how cytoskeleton perturbing drugs affect the activation of signalling molecules in combination with defined applications of physical loads to β1-integrins on the surface of MSC.

The activation of signalling pathways induced by mechanical forces share the signalling events which are stimulated by growth factors. We focus on the activation of two signalling proteins ERK and AKT to demonstrate how these signalling events depend on manipulation of the actin cytoskeleton when induced by a mechanical integrin stress. ERK is a MAP kinase and its activation and intracellular localization controls differentiation, proliferation and cell survival. AKT is a serine/threonine kinase and controls the PI3K-AKT signalling pathway. Similarly to ERK, it controls a broad range of cellular functions. Both signalling pathways are controlled by integrin mediated stimuli [8], [21], [22]. To correlate the effects of cytoskeleton perturbing drugs on signalling with the differentiation of MSC, we tested how the modulation of the actin cytoskeleton by these drugs controls parameters of osteogenic and adipogenic differentiation.

## Materials and Methods

### Ethics Statement

The study was approved by the Ethics Committee of the Rostock University Medical Centre (A 21/207). A written consent for using the samples for research purposes was obtained from all patients prior to surgery.

### Cells and culture

Human mesenchymal stem cells (MSC) were isolated from bone marrow which was obtained during median sternotomy during open heart surgery. According to a standard protocol, after density gradient centrifugation of the diluted marrow sample (d–1.077) interface enriched cells were cultured in expansion medium in 5% CO_2_ and at 37°C for three days. Adherent cells were harvested and grown in cell culture flasks using expansion medium for 14 days every three days before introduce them into the experiments. The purity of mesenchymal stem cells was proved by the absence of the hematopoetic marker CD34 and their ability to differentiate both to osteoblasts and adipocytes.

The following cell culture media were used during the experiments.

Expansion medium (EM) – Dulbecco's modified Eagle's medium (DMEM) containing 10% FCS (Gibco); osteogenic differentiation medium (ODM) – DMEM containing 100 nM dexamethasone, 10 mM β-glycerophosphate and 10 μg/ml ascorbic acid (all from Sigma-Aldrich, St. Louis, MO, USA); adipogenic differentiation medium (ADM) was obtained from Lonza (Basel, Switzerland) and supplemented with provided SingleQuots® containing 3-isobutyl-1-methylxanthine, dexamethasone, indomethacin, and insulin (Lonza). All media contained charge tested 10% fetal calf serum (FCS) (PAN-Biotech GmbH, Aidenbach, Germany) and 1% antibiotic-antimycotic solution (Invitrogen).

### Pharmacological treatment of cells

To perturb the actin cytoskeleton the following drugs were used in the experiments: Cytochalasin D (CytD) (0.5 µM), Latrunculin A (LatA) (0.01 µM, 0.1 µM) and Jasplakinolide Jasp) (0.01 µM) (all from Calbiochem, Merck, Darmstadt Germany). Prior to the experiments the cytotoxic effect of the drugs was tested in an MTT test to adjust the appropriate concentrations. In the experiments, drugs were diluted in DMSO and cells were cultured in a cell culture medium, containing the drugs for 24 prior to the experiments. As control, cells in culture medium, containing 0.1% DMSO were used. In the experiments to evaluate adipogenic and osteogenic differentiation, cells were cultured in the presence of the drugs for 7 days.

### Application of physical stress to integrins

For the experiments to apply physical integrin stress, cells were cultured in EM, containing reduced concentration of FCS (0.5%) 24 h prior and during the experiments. To stress integrin receptors mechanically, paramagnetic microbeads were coated with an antibody against the β1 integrin subunit. Preparation of beads and incubation are described elsewhere [23]. In brief, paramagnetic microbeads, 2.8 µm in size and sheep anti-mouse antibody coated (Dynal, Hamburg, Germany), were used. These beads were coated with a mouse anti-β1 integrin antibody (Beckman Coulter, Fullerton, CA, USA). After washing the beads in phosphate buffered saline (PBS), aliquots of the suspension were added to the cell monolayer and incubated for 30 min at 37°C. In average, 5-10 microbeads were attached to the β1 integrin subunit on the apical surface of one cell.

A magnetic device was used which has been described in detail earlier [23]. Briefly, the system consists of a coil system containing a ferrite core with two differently modelled poles to generate an inhomogeneous magnetic field. The average strength of the magnetic field between the magnetic poles was 0.015 T, as measured using a Hall probe.

A culture well containing the prepared cells (8,000 cells) was located between the two poles of the device. Drag forces act in the horizontal direction, i. e. in parallel to the apical cell surface, on the magnetic beads that are attached to the receptors. The forces subjected to one bead were adjusted to 2×10^−10^ N. A cyclic stress of 1 Hz (0.5 sec on, 0.5 sec off) was applied for 15 min. For comparison, cells were incubated with anti-β1 integrin antibody coated beads for 30 min for clustering and as control sample, the magnetic field was applied without beads for 15 min.

### Immunofluorescence

Cells cultured in EM on cover slips were fixed in 4% paraformaldehyde for 10 min, followed by permeabilization using 0.1% triton X-100. The actin cytoskeleton was visualized using FITC-labelled phalloidin (1∶100) (Sigma-Aldrich, St. Louis, Mo, USA). Vinculin was stained using mouse monoclonal anti-vinculin antibody (1∶100) (clone hVIN-1, Sigma-Aldrich), followed by incubation with a Cy3-conjugated rabbit anti mouse IgG antibody (1∶200) (Dianova, Hamburg, Germany) as secondary antibody.

The fluorescence images were analysed on a confocal laser scanning microscope Leica TCS SP2 (Leica Microsystems, Wetzlar, Germany) or LSM 780 (Carl Zeiss, Jena, Germany). For excitation of the fluorescence dyes an Argon Iron laser and a helium neon laser was used. Images of one confocal plane were analysed using the Leica software LAS AF Lite.

### Parameters of osteogenic and adipogenic differentiation

Osteogenic differentiation of MSC was tested by evaluation of the activity of alkaline phosphatase (ALP). After 7 days in ODM, containing one of the drugs, cells were washed in PBS and fixed in 4% paraformaldehyde (PFA) for 5 min. After washing again cells were incubated with 0.1% naphthol AS-MX phosphate and 0.1% fast red violet LB salt in a 2-amino-2-methyl-1,3-propanediol buffer (56 mM) for 10 min. As control, cells were cultured in ODM, containing 0.1% DMSO. Adipogenic differentiation of MSC was evaluated after 3 and 7 days in ADM, containing a drug. Fat containing cells were visualized using Bodipy staining. First cell nuclei were stained by incubation with Hoechst 33342 (diluted 1∶1000) (AppliChem, Darmstadt, Germany) followed by fixation in 4% PFA. Lipids were stained with BODIPY 493/503 (diluted 1∶250) (Molecular Probes, Carlsbad, CA, USA) for 15 min. Percentage differentiation towards adipocytes at day 3 was quantified by counting the number of cells containing droplets and dividing by total cell nuclei.

### Western blot analysis

Western blot analyses were performed for phospho-ERK. For controls, AKT and ERK were blotted. Briefly, adherent cells were lysed using the Bio-Plex™ cell lysis kit (Bio-Rad Laboratories, CA, Hercules, USA). For immunoblotting, 25 μg of total protein were separated by SDS-PAGE and then transferred onto PVDF membranes (Roche, Mannheim, Germany). The membranes were blocked and incubated overnight at 4°C with a rabbit monoclonal anti-phospho-p44/42 MAPK (ERK) (Thr202/Tyr204) (from Cell Signaling Technology, Danvers, MA, USA), anti-ERK (C16) (rabbit polyclonal antibody)(Santa Cruz Biotechnology), or anti-AKT (rabbit monoclonal antibody) (Cell Signaling Technology). As secondary antibody a HRP-conjugated monoclonal anti-rabbit IgG antibody (Dako, Glostrup, Denmark) was used. Protein expression was detected by chemiluminescence using ECL-substrate (Thermo Scientific, Rockfort, IL, USA). Immunoblots were repeated at least three times to ensure reproducibility. Blots were quantified by densitometry in a Gel Doc XR System (Bio-Rad, Hercules, CA, USA) and using the software Quantity One® and Image Lab™ (Bio-Rad).

### Bio-Plex Assay

The Bio-Plex technique (Bio-Rad) was used to quantitatively detect the expression of phospho-AKT. In brief, lysed cells were prepared using the Bio-Plex™ cell lysis kit and the protein content was measured using a Qubit® protein assay kit (Invitrogen, Karlsruhe, Germany). A 96 well plate was loaded with aliquots of protein. To quantify phospho-AKT the Bio-Plex S473 kit (Bio-Rad) was used to incubate cells with anti-phospho-AKT coated beads overnight. For the detection of the protein, a Bio-Plex phosphoprotein detection reagent kit (Bio-Rad) was used. The samples were then measured in an array reader Bioplex-200 system (Bio-Rad).

### Statistics

All experiments were repeated at least three times using MSC from three individual donors. To evaluate statistical differences, data received from stimulated cells were normalized to data of untreated control cells and results are presented as mean values and standard deviation. Significant differences were tested by one-way ANOVA and multiple comparison using SPSS 15.0 software (SPSS Inc., Chicago, IL, USA). Significance levels were set at p≤0.05 or p≤0.01.

## Results

**Figure 1:**
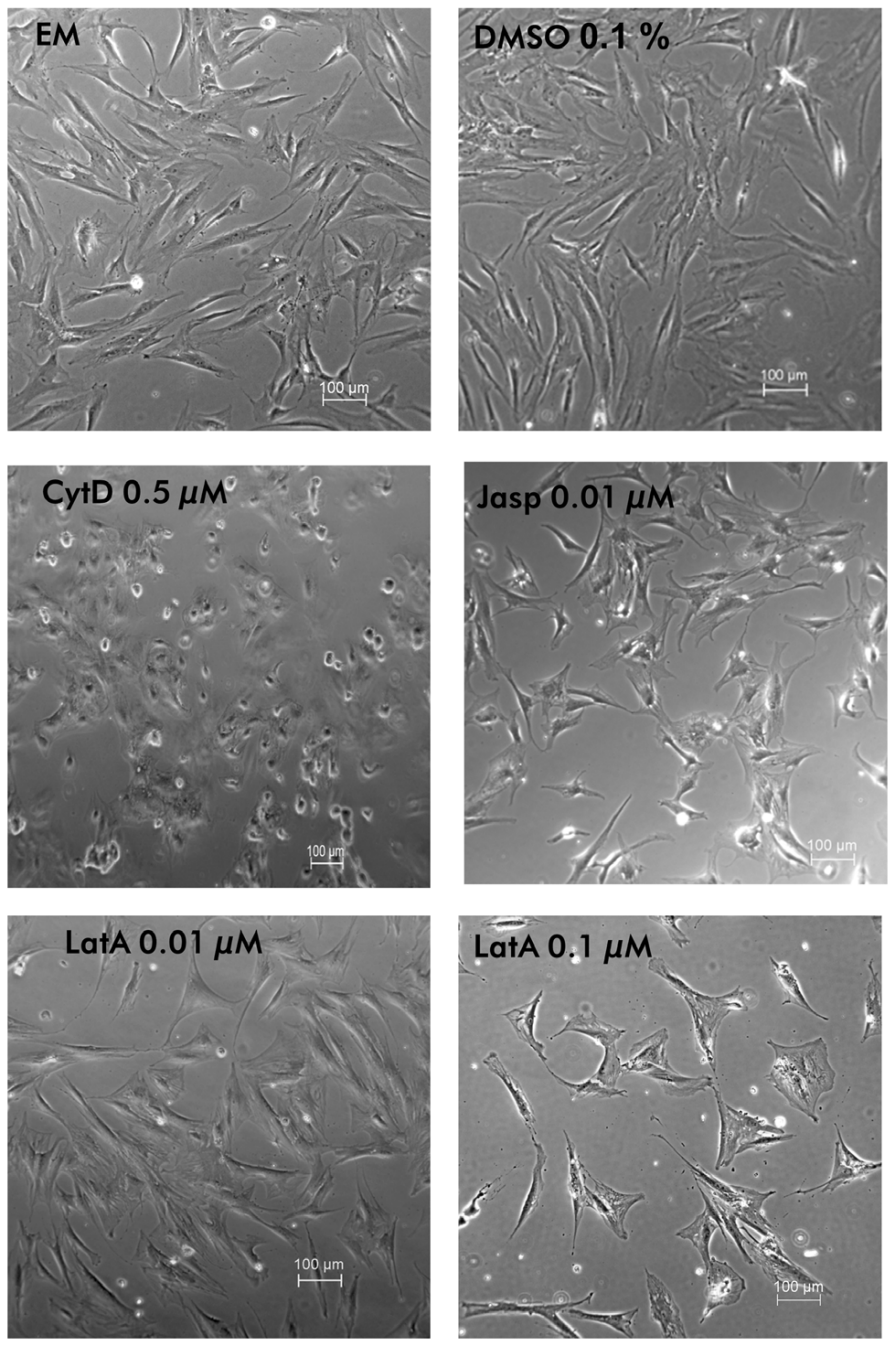
Morphology of MSC. In light microscopy, morphology of MSC cultured for 24 h in expansion medium (EM), containing cytoskeleton perturbing drugs at the indicated concentrations was studied. All drugs induced differential changes in the cell shape. DMSO alone did not induce alterations of the cell morphology.

The study was aimed to modify the actin cytoskeleton of MSC without impairing the survival of the cells. Therefore, loss of cell adhesion and reduced metabolic activity tested in the MTT test were used to reveal the critical concentration for the application of the drugs to manipulate the actin network but maintain cell survival (data not shown). First we were interested in the effects of the three pharmacological agents on the cell shape (Fig. 1). DMSO in the culture medium, which was required to dissolve the drugs, did not affect cell morphology. CytD induced a marked change in cell shape. Cells converted from spindle shaped to more round cells with similar length and width. LatA induced a broader cell shape, obviously visible at the higher concentration of 0.1 µM. Jasp did not provoke an obvious change in the cell morphology at a concentration of 0.01 µM, but some cells became retracted. Higher concentrations impaired the cell survival.

**Figure 2:**
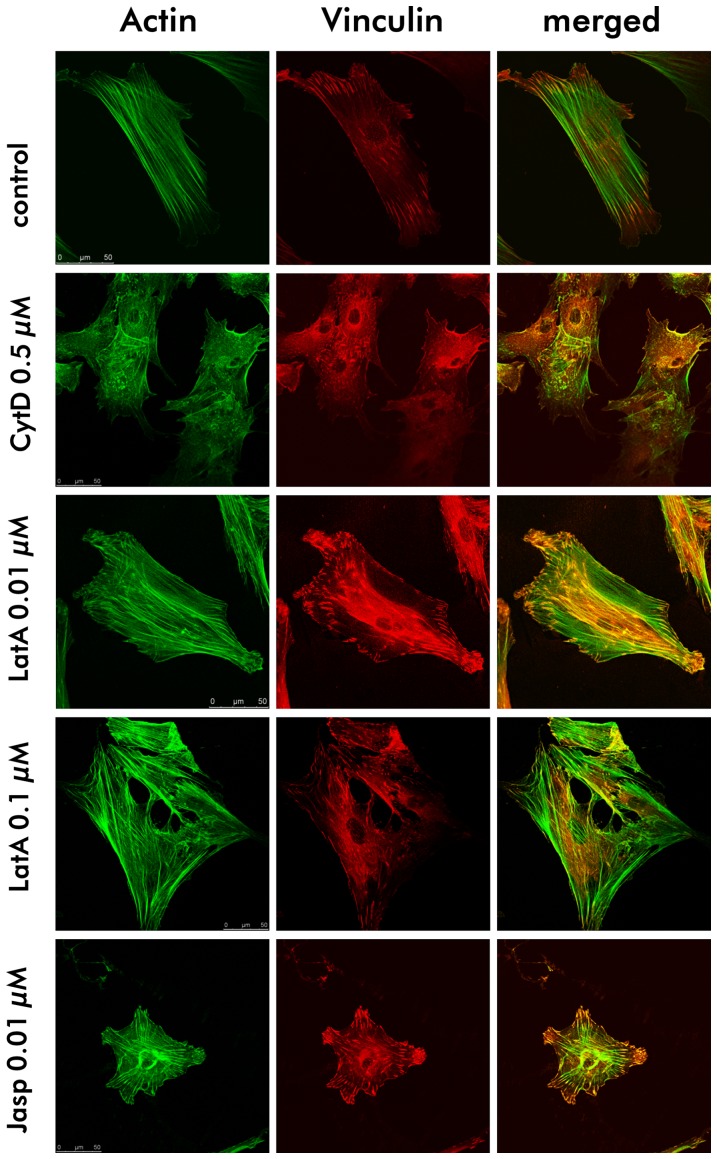
Changes in the cytoskeletal organization. Fluorescence images of the actin cytoskeleton (green) and vinculin (red) of MSC, cultured for 24 h in EM containing drugs at the indicated concentrations. All drugs induced changes in the cytoskeletal organization and in some cases colocalization between actin and vinculin was abrogated.

Next, we explored the organisation of the actin cytoskeleton and its colocalization with the focal adhesion protein vinculin by fluorescence staining of the two cellular components (Fig. 2). Control cells in DMSO containing medium revealed distinct actin filaments which are partly organized in parallel and colocalize with vinculin at the ends of the filaments. Incubation with CytD induced a breakage of the filaments into smaller pieces and the colocalization with vinculin was abrogated. LatA at a concentration of 0.1 µM induced a more irregular organisation of the cytoskeleton, however the filaments were maintained and at lower concentration a colocalization with vinculin is visible. When cells were incubated with Jasp, distinct actin filaments are expressed which are well organized and colocalize with vinculin at the end of the cell extentions. It was obvious that actin formed a ring-like, strongly expressed structure around the nucleus.

**Figure 3:**
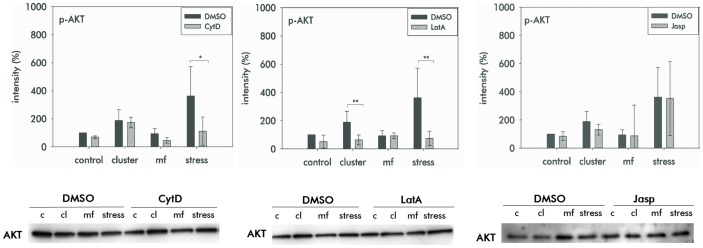
Activation of the signalling protein AKT. Quantitative analyses of phosphorylated AKT (p-AKT) in MSC after application of mechanical stress to β1-integrin and in the presence of CytD (0.5 mM), LatA (0.1 mM) or Jasp (0.01 mM). For controls MSC were cultured in EM containing 0.1% DMSO (DMSO). Measurements were performed using a Bioplex assay. For control of the protein content, AKT was blotted in Western blot (below). p-AKT induced by mechanical load was inhibited by CytD and LatA, but not by Jasp. The intensities are relative to the control. Control (c) – cells without mechanical stress and no magnetic field, cluster (cl) – integrins were clustered by incubation with antibody coated beads, mf (magnetic field) – cells were exposed to the magnetic field without beads, stress – integrins were mechanically loaded by drag forces. (Results are mean values of three independent measurements, asterix indicate statistical significance at p≤0.05* or p≤0.01**).

**Figure 4:**
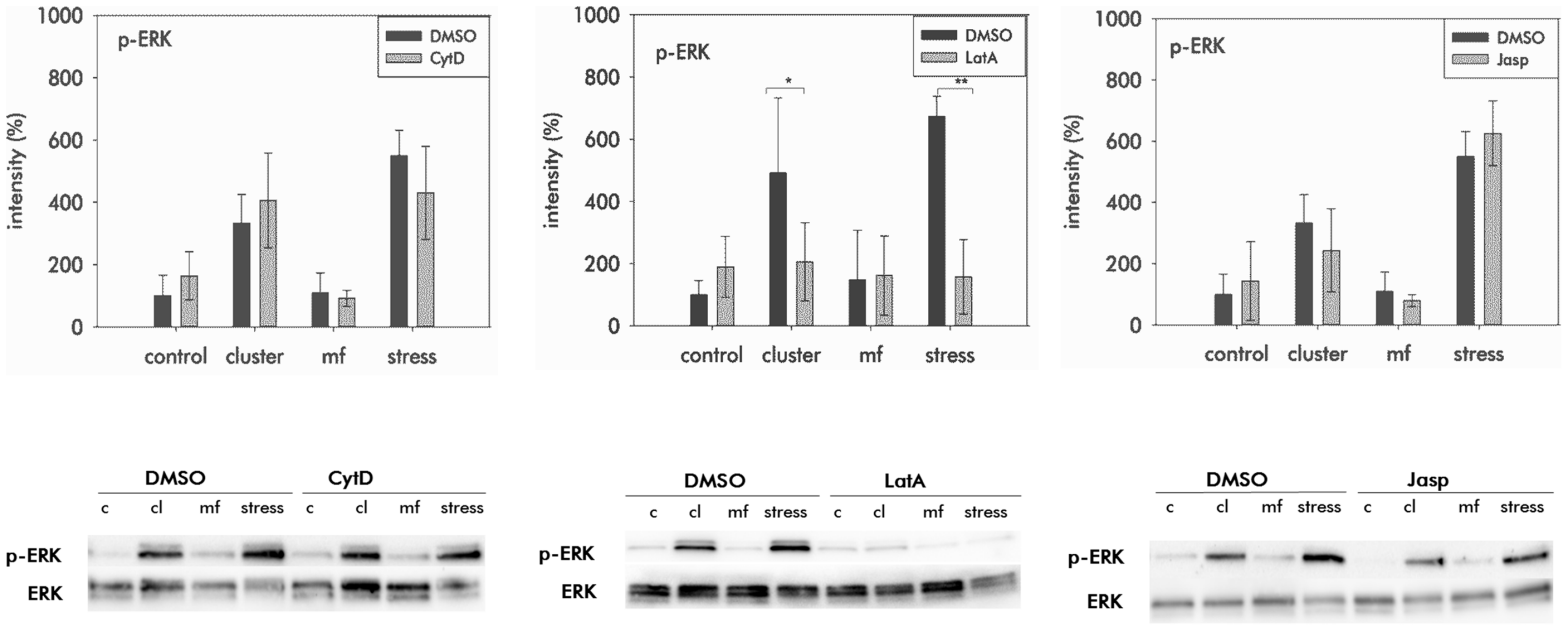
Activation of the signalling protein ERK. Quantitative analyses of phosphorylated ERK (p-ERK) in MSC after application of mechanical stress to β1-integrin and in the presence of CytD (0.5 mM), LatA (0.1 mM) or Jasp (0.01 mM). For controls MSC were cultured in EM containing 0.1% DMSO (DMSO). Measurements were performed using Western blot followed by quantitative densitometric analyses. Representative blots for p-ERK and ERK are shown below. p-ERK induced by mechanical load was inhibited by LatA, but not by Cyt D and Jasp. The intensities are relative to the control. Control (c) – cells without mechanical stress and no magnetic field, cluster (cl) – integrins were clustered by incubation with antibody coated beads, mf (magnetic field) – cells were exposed to the magnetic field without beads, stress – integrins were mechanically loaded by drag forces. (Results are mean values of three independent measurements, asterix indicate statistical differences at p≤0.05* and p≤0.01**).

After having seen that all three drugs induced changes in cell shape and in the organization of the cytoskeleton we were interested, whether modifications of the actin cytoskeleton by the three drugs affect the activation of the signalling proteins ERK and AKT by mechanical forces applied to integrins. In all the controls that we used for comparison with cells under the influence of drugs, mechanical forces provoked a phosphorylation of ERK and AKT (Fig. 3, 4). This was observed when antibody coated beads were incubated to cluster integrins and the effect was increased when drag forces were applied to integrins. Thus, integrin mediated mechanotransduction provokes a signalling which involves the activation of ERK and AKT. Application of the magnetic field alone, which served as another control had no effect on activation of AKT and ERK. After preincubation of the cells with CytD we found that activation of AKT due to applied forces to integrins was reduced (Fig. 3). In contrast, activation of ERK induced by a mechanical integrin stress was not impaired by CytD (Fig. 4). The minor activation of ERK and AKT due to integrin clustering remained unaffected by CytD. When LatA were added to the cells, a complete inhibition of the activation of both AKT and ERK was measured (Fig. 3, 4). This concerns both the inhibition of phosphorylation due to the application of drag forces and due to integrin clustering. In contrast to the effects of the depolymerising drugs, the actin stabilizing drug Jasp did not affect the activation of both signalling proteins ERK and AKT due to mechanical forces (Fig. 3, 4). This concerns the activation during integrin clustering and after application of drag forces.

**Figure 5:**
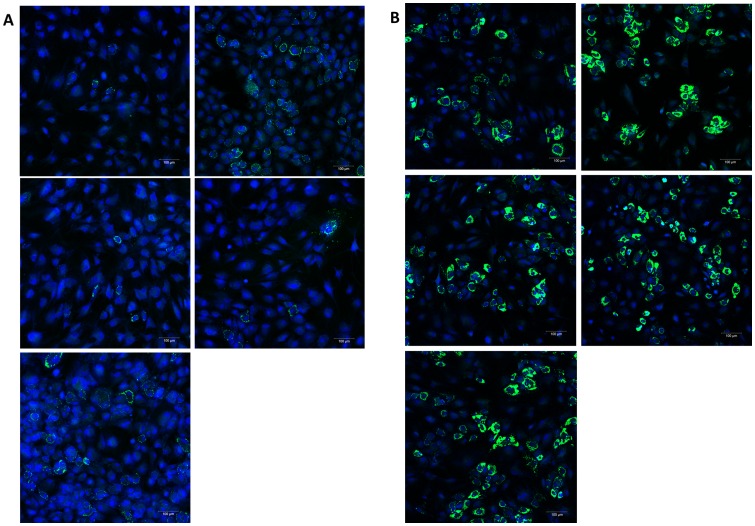
Adipogenic differentiation of MSC. Adipogenic differentiation of MSC in the presence of the cytoskeleton perturbing drugs CytD, LatA and Jasp. Adipogenic differentiation was evaluated by analysing the uptake of fat droplets by the cells (fat droplets – green, nuclei – blue). A. Day 3 in adipogenic differentiation medium. Suboptimal uptake of fat droplets but increased number of fat containing cells in the presence of CytD and Jasp (percentage of fat containing cells: DMSO –3%, CytD –26%, Lat 0.01–3%, Lat 0.1–7%, Jasp –23%). B. Day 7 in adipogenic differentiation medium. Optimal uptake of fat droplets without differences in the number of fat droplets containing cells due to the drug treatment.

**Figure 6:**
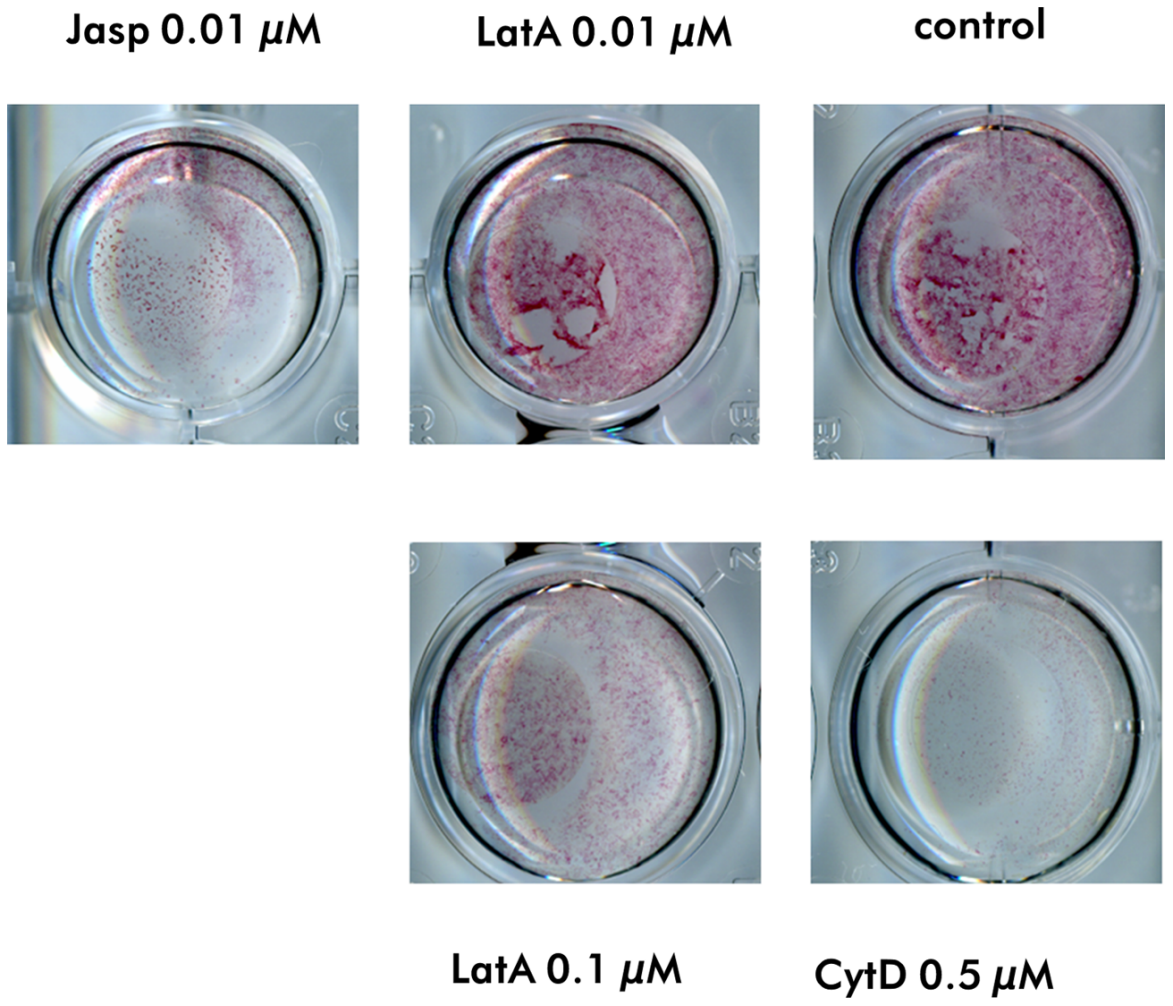
Osteogenic differentiation of MSC. Osteogenic differentiation of MSC in the presence of the cytoskeleton perturbing drugs CytD, LatA and Jasp. Osteogenic differentiation was evaluated by staining ALP activity (red staining) after 7 days in osteogenic differentiation medium (ODM). All three drugs inhibited the activation of ALP.

To see, whether the modifications of the cytoskeleton which affected the activation of signalling molecules provokes functional consequences in mesenchymal stem cells, we studied markers of adipogenic and osteogenic differentiation. When the cells were cultured in adipogenic differentiation medium, within 7 days the cells contained lipid droplets, which indicates the differentiation to fat cells (Fig. 5B). At this time no differences were found in the number of fat droplet containing cells between controls and cells treated with drugs. However, at day 3 both the depolymerizing drug CytD and the stabilizing drug Jasp induced an increased number of adipocytes compared with the control, which indicates a faster differentiation to adipocytes (Fig. 5A). LatA at both concentrations did not induce an enhanced adipogenic differentiation. To evaluate the effect of cytoskeletal modifications on osteogenic differentiation, the drugs were added for 7 days to MSC cultured in osteogenic differentiation medium. A pronounced decrease of the ALP activity as a marker for osteogenic differentiation we observed with all three cytoskeletal modulators (Fig. 6). Similar strong effects were observed with CytD as an inhibitor of the cytoskeletal polymerization and with Jasp, which promotes actin polymerization. The two concentrations of LatA revealed a dose dependency with a distinct effect on activation of ALP activity with 0.1 µM LatA.

## Discussion

As a control result we demonstrated that a short time mechanical load to β1-integrin induced an activation of ERK and AKT in MSC. In addition, in earlier studies we have shown that this integrin load provokes signalling events like an intracellular calcium signal and a physical anchorage of integrins to the cytoskeleton [24], [25]. Recently, the actin-binding protein filamin A was identified as molecular link between integrin and the cytoskeleton [26]. Strain increases β-integrin binding to filamin A and facilitates mechanotransduction. Thus, these results together support the mechanical coupling between integrins and the cytoskeleton as well as reveal further signalling events when mechanically loading of integrins on the surface of cells without obvious changes in the cell shape. We now demonstrate that application of three agents which modulate the actin cytoskeleton affects differentially the activation of ERK and AKT induced by a physical stress to integrins. While the actin inhibiting drugs CytD and LatA reduced the phosphorylation of the signalling proteins to different extents and in dependence on the type of the protein, the actin stabilizing agent Jasp did not affect the mechanically induced activation of both ERK and AKT. CytD induced dramatic changes in cell morphology which was accompanied by a partly fragmentation of the cytoskeletal filaments and loss of a colocalization between vinculin and actin. This correlates with an impaired activation of AKT due to CytD treatment, but despite the distinct loss of structural organization of the cytoskeleton, CytD had surprisingly no obvious effect on ERK activation. Inhibition of activation of AKT by CytD was also shown during cyclically stretching of mouse embryonic stem cells on a flexible culture plate [27]. With our approach to apply a mechanical load and to explore the effect of drugs on the cytoskeleton we could now establish the direct link to the integrins as transducers of the mechanical load. The finding that ERK activation was not inhibited by CytD in our experiments correlates with studies using other cells including cardiomyocytes, in which stretch was applied using a silastic membrane [28]. The authors found that pretreatment with CytD did not affect ERK activation, but prevented its nuclear translocation. Activation of ERK by integrins may be facilitated by alternative pathways, e. g. the signalling proteins Fyn and Shc can be involved instead of the focal adhesion kinase FAK [29]. However, our result that LatA completely blocked the phosphorylation of ERK due to a mechanical integrin load indicates that the actin cytoskeleton is required for the activation of ERK in this context. Concerning the mechanisms, how LatA and CytD perturb the actin cytoskeleton, it is known that both drugs sequester actin monomers to prevent polymerization [30]. CytD caps the barbed end of actin filaments, whereas LatA binds to actin at the nucleotide binding cleft and in vitro forms a nonpolymerizable complex with G-actin [31], [32]. More specifically both drugs inhibit the movement of mDia, which belongs to the class of formins, on actin filaments [33]. The speed of the mDia movement correlates with actin elongation rates. Mechanical stimuli regulate the activation of mDia by modulating the concentration of G-actin [26]. The differences we observed between LatA and CytD concerning the effect on ERK activation may be explained by additional activities. For example, LatA is able to prevent specific binding of thymosin-β4 to the actin cytoskeleton, which in complex with profilin regulates the dynamics of the cytoskeleton [32], [34]. In contrast to both inhibitors of actin polymerization, the actin stabilizing drug Jasp did not affect activation of both AKT and ERK due to mechanical integrin stress. Jasp stabilizes actin filaments and is a potent inducer of actin polymerization [35]. In our experiments Jasp induced a ring like distribution of actin around the nucleus and strong filaments directed to the filopodia. As shown in the fluorescent image, cells may be retracted, which is supported by a previous study at higher concentrations of Jasp [31]. However, despite these distinct alterations in the structural organization of the actin cytoskeleton by Jasp, the integrin mediated mechanically induced activation of both signalling molecules was not affected.

To correlate the observed effects of drugs on AKT and ERK with parameters of cell differentiation we found that ALP activation as a marker for osteogenic differentiation was impaired by all three drugs, which suggests that an intact cytoskeleton is required. This finding is supported by a study demonstrating that spontaneous osteogenic differentiation of MSC on 3D-microcarriers was dependent on cytoskeletal tension and actomyosin contraction [36]. Although Jasp did not affect activation of AKT and ERK, also this drug impaired the osteogenic differentiation. This suggests that in addition to activation of both signalling proteins other cytoskeletally associated mechanisms are relevant for osteogenic differentiation. In endothelial progenitor cells of the rat, the actin stabilizing effect of Jasp impaired several functions, including migration and proliferation [37]. In vivo Jasp impaired the capacity of these cells to reendothelialize vessels. Previous studies provided evidence that mechanisms involved in MSC differentiation depend on the cell shape that is regulated by the cytoskeleton [18]. A spread phenotype of MSC or an elongation directed by grooves and grids favour an osteogenic differentiation [18], [38]. The phenotype changes we observed due to the drugs compared with the control was the formation of a more rounded cell type due to CytD and a broader cell shape with LatA and Jasp, which might be of relevance. Our examination of the adipogenic differentiation revealed that already after three days an adipogenic phenotype was detected after CytD and Jasp treatment compared with the control and treatment with LatA. As shown earlier, poorly spread MSC differentiate to adipocytes [18]. Such a round shape to favour adipogenesis can be generated by small ECM islands or as we and others have shown by treatment with CytD which inhibits cytoskeletal tension [39]. There is evidence that the ERK pathway is essential in different steps of the adipogenic differentiation of stem cells [40]. In our experiments, only LatA blocked the activation of ERK, which may be a reason why fat droplets were not detected in the presence of LatA after three days in adipogenic medium.

## Conclusions

Our results provided insights into the role of the actin cytoskeleton in integrin mediated mechanically induced signalling pathways in MSC. Two actin depolymerizing drugs and one actin stabilizing agent affected differentially the mechanically induced activation of AKT and ERK. This indicates that various mechanisms associated with the cytoskeleton are involved in the mechanical control of signalling events. In addition to the effects on activation of signalling molecules, the actin perturbing drugs affected parameters of osteogenic and adipogenic differentiation of MSC. Adipogenic differentiation can be promoted by cytoskeletal drugs and osteogenic differentiation was inhibited by drugs. Together, actin filament perturbing drugs are suitable to explore molecular mechanisms in the biological response of MSC. In addition, non-toxic cytoskeleton modulating drugs are promising candidates to regulate cellular functions of stem cells.
